# Adult dural arteriovenous fistulas in Galen region: More to be rediscovered

**DOI:** 10.3389/fneur.2022.957713

**Published:** 2022-10-28

**Authors:** Tianqi Tu, Zihao Song, Yongjie Ma, Chengbin Yang, Xin Su, Chuan He, Guilin Li, Tao Hong, Liyong Sun, Peng Hu, Peng Zhang, Ming Ye, Hongqi Zhang

**Affiliations:** Department of Neurosurgery, Xuanwu Hospital, Capital Medical University, Beijing, China

**Keywords:** dural arteriovenous fistula, Galenic area, endovascular management, cognitive dysfunction, progressive dementia

## Abstract

**Background:**

Dural arteriovenous fistulas (DAVFs) in the Galen region are the most deeply located and most complex type of dural arteriovenous fistulas. However, cases of DAVFs in this region have not been well described. Thus, we aimed to summarize the characteristics of Galenic DAVFs involving clinical symptoms, anatomical architecture, and drainage patterns, providing experientially therapeutic strategies for these lesions based on our 20 years of clinical experience.

**Methods:**

We retrospectively examined 31 patients with Galenic DAVFs between January 2000 and June 2021. A comprehensive analysis was carried out based on the symptoms, imaging features, feeding arteries, draining veins, number and location of the fistulas, choice of treatment methods, and prognosis assessment.

**Results:**

Twenty-nine patients received endovascular embolization, and no perioperative deaths occurred. A transarterial approach was performed in 27 patients, and a combined transarterial and transvenous approach in one. And in one case, access was established by surgical drilling and embolization was done via the venous route. Twenty-four cases were completely obliterated after first embolization, and another five cases received a second period treatment. Only one patient developed cognitive dysfunction after embolization, and the outcomes of the remaining patients were improved at long-term follow-up.

**Conclusion:**

The understanding of symptoms of non-hemorrhagic neurological deficits in DAVF needs to be further clarified. Lesions with pial feeders may be considered first when determining surgical orders. Multi-approach and multi-stage embolization would be safe and effective. Excessive embolization and deep-vein system obstruction should be avoided. Approach creation by surgery would be an innovative interventional therapy.

## Introduction

Dural arteriovenous fistulas (DAVFs) consist of abnormal arteriovenous interconnections in or around dural venous sinuses, as well as the meningeal veins or intradural pial veins (1–3). DAVFs account for 10–15% of all cerebrovascular malformations, which is one of the important causes of cerebral hemorrhage and neurologic deficits (1, 3). Given that they are positioned in the most complex and the deepest anatomical position, DVFs located in the Galenic region in adults (aGDAVFs) are uncommon.

Deep intracranial neurovascular relationships, especially the veins near the pineal and corpora quadrigemina region are the most complex. Deep cerebral veins, including the internal cerebral vein (ICV), the basal vein of Rosenthal (BVR) and their branches, mainly inflow to the vein of Galen, forming venous intersections and draining the deep venous system (DVS) (4). Reflux obstruction or venous hypertension in the drainage vein due to fistula formation can lead to specific and progressive symptoms. Non-hemorrhagic neurological deficits should be fully recognized for DAVFs (5). Accordingly, these symptoms need to be distinguished from cognitive dysfunction caused by degenerative diseases, otherwise misdiagnosis would lead to constant threats.

In this article, we present our interventional experience with aGDAVF treatment to evaluate angiographic findings, define its characteristics, and provide effective treatment strategies.

## Methods

We retrospectively examined 31 patients with aGDAVFs who received treatment at the neurosurgery department of our hospital between January 2000 and June 2021. Retrospective analysis was carried out based on gender, age, initial and concomitant symptoms, imaging features, feeding arteries, draining veins, number and location of the fistulas, embolic strategies, and prognosis assessment.

## Results

### Patient profiles

In total, 31 patients were included in this work from January 2000 to June 2021, and a predominance in gender was revealed, for 25 men and six women were documented. The age range was from 23 to 73 years old.

### Clinical presentation

Four patients suffered hemorrhagic neurological deficits, two of whom presented with subarachnoid hemorrhage (SAH, patients 5 and 16), and another two suffered intracerebral hemorrhage (ICH, patients 8 and 25). Clinical manifestations of cognitive impairment involving memory deterioration, computation ability impairment, lags in response, and mutism were accompanied by eight patients. Other symptoms such as headaches (12/31), tinnitus or cranial murmurs (4/31), ophthalmic signs (2/31), and limb weakness (9/31) were common in DAVF, and were also important factors influencing patients' health-related quality of life. Four patients had no obvious symptoms, and were found accidently through routine physical examination.

The main clinical manifestations are summarized in Table 1.

**Table 1:** Clinical presentations, angiographic and clinical outcomes and MRS scores.

**Case No**.	**Presentation**	**Cognition impairment**	**Cerebral hemorrhage**	**Clinical outcome**	**MRS score**	
					**Pre-**	**Late-**
1	Asymptomatic	No.	No.	Asymptomatic	0	0
2	Tinnitus (right side)	No.	No.	Subsequently asymptomatic	1	0
3	Headache	No.	No.	Asymptomatic	1	0
4	Headache	No.	No.	Asymptomatic	1	0
5	Headache(SAH), Depression	No.	SAH	Subsequently asymptomatic	5	1
6	Headache, tinnitus (left side)	No.	No.	Asymptomatic	2	0
7	Conjunctival hyperemia, ocular protrusion (right side)	No.	No.	Ocular symptoms disappeared.	2	0
8	Cognitive impairment	Yes.	ICH	Previous symptoms relieved	5	2
9	Cognitive impairment, unsteadily walk	Yes.	No.	Previous symptoms relieved	4	1
10	Cognitive impairment, unsteadily walk	Yes.	No.	Previous symptoms relieved	5	3
11	Cognitive impairment, vision loss (left side)	Yes.	No.	Previous symptoms relieved	3	1
12	Cognitive impairment	Yes.	No.	Previous symptoms relieved	4	3
13	Cognitive impairment, memory deterioration, unsteadily walk	Yes.	No.	Previous symptoms relieved	5	2
14	Cognitive impairment, weakness of left limb	Yes.	No.	Previous symptoms relieved	5	2
15	Headache, intracranial murmurs, weakness of left limb	No.	No.	Previous symptoms remained.	4	3
16	Episodic headache with vomiting	No.	SAH	Previous symptoms relieved	3	0
17	Headache, intracranial murmurs	No.	No.	Asymptomatic	2	0
18	Headache, unsteadily walk	No.	No.	Asymptomatic	3	0
19	Asymptomatic	No.	No.	Asymptomatic	0	0
20	Headache	No.	No.	Previous symptoms relieved	3	1
21	Headache	No.	No.	Subsequently asymptomatic	3	1
22	Headache	No.	No.	Asymptomatic	3	0
23	Weakness of left limb	No.	No.	Asymptomatic	3	0
24	Asymptomatic	No.	No.	Asymptomatic	0	0
25	Sudden unconsciousness	No.	ICH	Subsequently asymptomatic	5	2
26	Unsteadily walk	No.	No.	Subsequently asymptomatic	2	0
27	Asymptomatic	No.	No.	Asymptomatic	0	2
28	Headache	No.	No.	Subsequently asymptomatic	1	0
29	Cognitive impairment, weakness of lower limbs	Yes.	No.	Previous symptoms relieved	3	2
30	Asymptomatic	No.	No.	Asymptomatic	0	0
31	Numbness of left upper limb	No.	No.	Subsequently asymptomatic	1	0

### Angiographic architecture

DAVFs in the Galenic region are complex, for they are often supplied by multiple arterial systems (external carotid system, internal carotid system, and vertebral artery system). As we documented in all 31 cases, 24 received blood supply from all systems simultaneously, six were supplied by two systems at least, and only one was supplied by the external carotid system. The dominant suppliers were from external carotid artery (ECA) system, as middle meningeal artery (MMA) (24/31) and occipital artery (OA) (20/31) were the main feeders. Other suppliers involving superficial temporal artery (STA) (7/31 patients, 22.6%), ascending pharyngeal artery (APA) (1/31) and internal maxillary artery (IMA) (2/31), together with their miscellaneous branches (1/31) contributed to a lesser degree. Feeding arteries originating from the internal carotid artery (ICA) system mainly included the meningohypophyseal trunk (MHT) (12/31), ophthalmic artery (OphA) (4/31 patients), pericallosal artery (2/31), anterior choroidal artery (AChA) (1/31), as well as ICA branches (1/31 patients) also participated in this kind of blood supply. Posterior meningeal artery (PMA) (7/31), posterior cerebral artery (PCA) (13/31) and superior cerebellar artery (SCA) (11/31) were the common feedings from the vertebral artery (VA) system, and a few feeders also originated from VA branches (6/31), the posterior medial choroid artery (MPChA) (4/31), and posterior lateral choroid artery (LPChA) (1/31).

Enlarged drainage veins with venous lake formation were typical angiographic features, since huge venous lakes in 18 patients of the 31 could be clearly seen *via* angiography. Supratentorial drainages were the main outflow routes, involving ICV, BVR and internal occipital vein, etc. The straight sinus was also an important outflow tract, reaching the transverse sinus, sigmoid sinus. Fistulas with cortical venous drainage could also drain into the superior sagittal sinus. Only one fistula drained by superior veins of cerebellar hemisphere (the patient 19).

Detailed angiographic architectures and interventional features were listed in Table 2.

**Table 2:** Angiographic characteristics and treatment technique.

**Case No**.	**Feeders**	**Draining veins**	**Cornard classification**	**Venous lake**	**Main approaches**	**Embolic agents**	**Treatment Stage**
1	L PMA, R MMA, L MHT	Straight sinus, superior sagittal sinus	IIa+b	No	R MMA	ONYX 18	1
2	R MHT, Bi MMAs, Bi STAs, Bi OAs, L PChA, L SCA	Straight sinus, VoG-BVR-cavernous sinus	IIa+b	Yes	R ICA, R ECA	ONYX 18	1
3	R OphA, L MMA, L PMA, L OAs	Tentorial sinus, falcine sinus, suboccipital venous plexus	IIa+b	Yes	L MMA, L PMA	NBCA	1
4	Bi OphAs, Bi PMAs, Bi OAs, Bi MMAs, L SCA	Straight sinus, ICV	IIa	Yes	L OphA, R PMA, L OA, Bi MMAs	ONYX 18	1
5	Bi LPChAs, L OA, L PMA	Straight sinus	IIa	No	L PCA	ONYX 18	1
6	Bi MHTs, R ECA, R MMA, L PChA, Bi PCAs	Straight sinus, superior sagittal sinus	IIa+b	Yes	L PChA, Bi MMAs, R PICA	ONYX 18+ Glubran+ Coils	2
7	L MHT, Bi MMAs, L IMA, Bi PCAs, Bi OAs, L PMA, Bi SCAs,	Straight sinus, VoG-BVR-cavernous sinus-sylvian vein and angular vein	IIa+b	Yes	L MMA, L angular artery	ONYX 18 +NBCA+Coils	2
8	R MMA, L SCA	Straight sinus, superior sagittal sinus	IIa+b	No	R MMA	NBCA	1
9	Bi OAs, R MMA, L SCA, R pericallosal artery	Straight sinus, superior sagittal sinus	IIa+b	Yes	R MMA	ONYX 18	2
10	R MMA	ICV, BVR, falcine sinus	IIa	No	R MMA	ONYX 18	1
11	Bi MHTs, Bi MMAs, R PCA	Straight sinus, BVR	III	Yes	Bi MMAs	ONYX 18	1
12	R PCA, R OA, R MMA	ICV, BVR, cortical vein of occipital lobe	IIa+b	No	R PCA, Bi MMAs	ONYX 18	1
13	L ECA	ICV, BVR, straight sinus	IIa+b	No	L STA	ONYX 18	1
14	Bi MMAs, Bi STAs, Bi OAs, Bi MHTs, Bi VAs	Straight sinus, superior sagittal sinus	IIa+b	Yes	R ECA, L MMA	ONYX 18+ONYX 34	2
15	Bi ICAs, Bi ECAs, Bi VAs	Straight sinus, ICV-BVR-cavernous sinus	IIa+b	Yes	-	-	-
16	R MHT, Bi MMAs, Bi OAs, R STA, Bi PCAs	Straight sinus, falcine sinus	IIa	No	R LPChA, R MMA, L SCA	NBCA	1
17	Bi MMAs, Bi OAs, Bi STAs, L APA, L OphA, L MHT	Straight sinus	IIa+b	Yes	Bi MMAs, Bi OAs, L APA	ONYX 18+Glubran	1
18	Bi SCAs, L MPChA	BVR-cavernous sinus, sylvian vein-superior sagittal sinus	IIa+b	No	R SCA, L MPChA	ONYX 18	1
19	Bi SCAs, L PCA, L VA, L OA	ICV, BVR, veins of cerebellar hemisphere	IIa	No	-	-	-
20	L MPChA, Bi MMA, branches of L VAs	Straight sinus, superior sagittal sinus	III	Yes	L MPChA, R MMA	Glubran	1
21	Bi MMAs, Bi OAs, L pericallosal artery, L SCA	Straight sinus, superior sagittal sinus	IIa+b	Yes	L ACA, L MMA	ONYX 18	1
22	Bi OAs, Bi MHTs, Bi PCAs, Bi SCAs	Straight sinus	IV	No	R OA	ONYX 18	1
23	Bi MMAs, Bi OAs, R AchA, Bi PMAs, Bi PCAs, L SCA	Straight sinus	IV	Yes	L PMA, L SCA, L PCA, Bi OAs, Bi MMAs, Bi MPChAs	ONYX 18	2
24	Bi MHTs, Bi OAs, Bi MMAs, Bi PCAs, Bi MPChAs	Straight sinus	IV	No	L MPChA, R ECA	ONYX 18+Glubran +balloon	1
25	R MMA, Bi OAs, Bi PCAs	Straight sinus	III	No	Bi PCAs, R MMA	Glubran	1
26	Bi PCAs, L MMA, L STA, L OA	Straight sinus	III	Yes	R PCA, R MPChA	ONYX 18+Glubran	1
27	Bi MMAs, Bi OAs, Bi MHTs, R PMA,L IMA, L MPChA	Straight sinus, BVR	IIa	Yes	L MPChA, L MMA	ONYX 18+Glubran	1
28	Bi STAs, Bi OAs, R MMA, R OphA, Bi PCAs, Bi SCAs	Straight sinus	III	Yes	R MMA	ONYX 18	1
29	Bi MMAs, Bi PCAs, R STA	Straight sinus	IIa	Yes	R MMA	ONYX 18	1
30	Bi MHTs, Bi MMAs, Bi VAs	Straight sinus	IIa+b	Yes	L MMA	ONYX 18+Coils	1
31	L OA, L VA	Straight sinus, ICV	IIa	No	R OA	ONYX 18	1

### Treatment strategies

For exhaustive evaluation of anatomic architecture, total cerebral angiography should be done through VA, ICA, and ECA of both sides.

**Figure 1:**
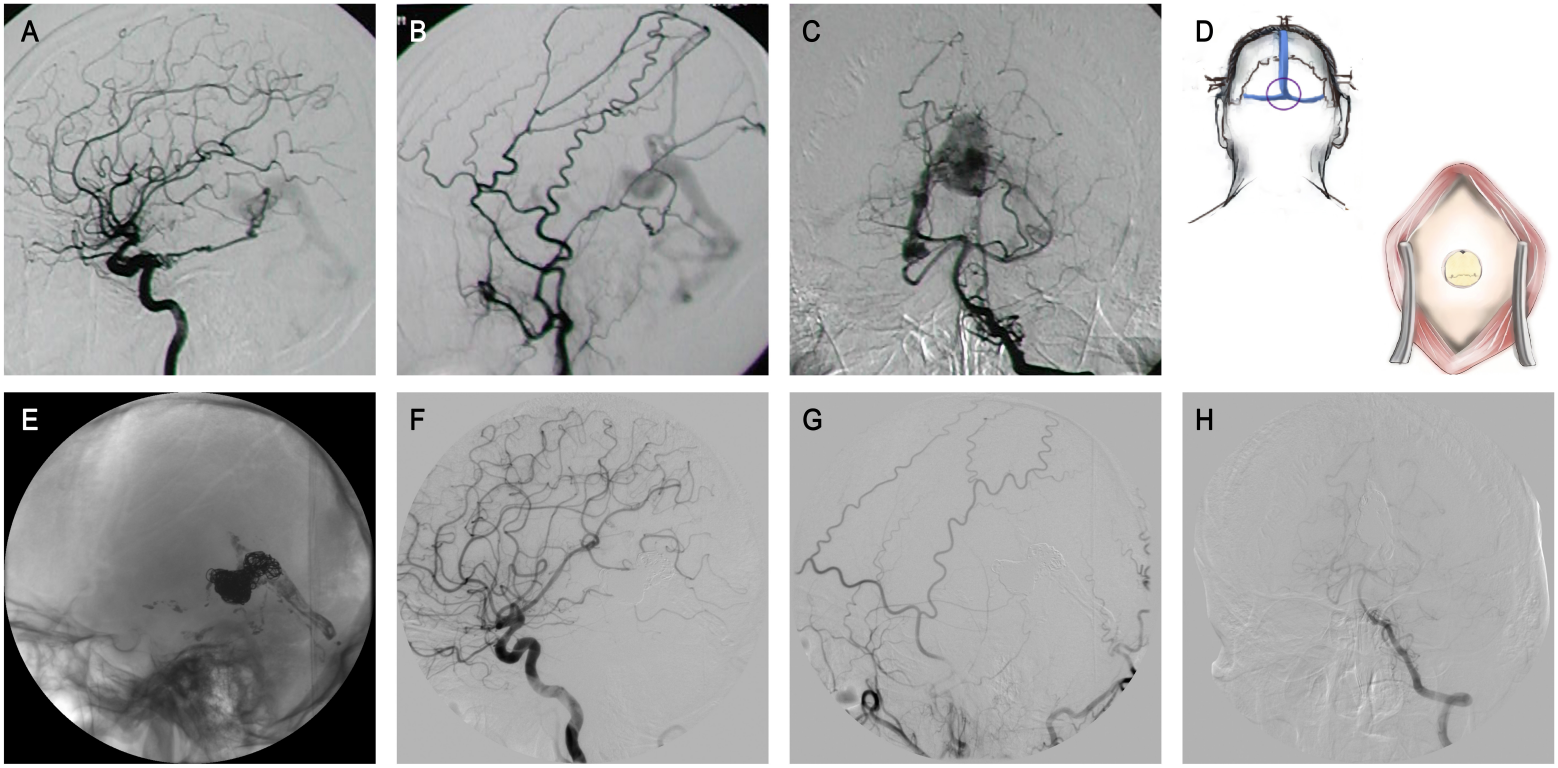
The patient 9 presented with a history of conjunctival congestion for 5 years and proptosis of the right eye for 3 years. **(A–C)** Lateral ICA and ECA angiography, and anteroposterior VA angiography revealed GDAVF feeded by left IMA, bony branch and parietal occipital branch of bilateral MMA, bilateral PCA and OA, left PMA, and bilateral SCA. **(D)** Schematic diagram. A hole was drilled surgically at the external occipital protuberance to establish an appraoch. **(E–H)** DSA images obtained 6 months after treatment showed almost completely fistula embolization.

Then, the following embolic strategies can be considered. First, priority should be given to obliterate pial feeders with soft microcatheters. Second, to place the catheter in the optimum position, surgical pathways could be established (as the patient 7, described in Figure 1). Thirdly, DMSO-based liquid embolic agents might be the main choice, while the agent's polymerization speed and dispersion properties should be considered. Any excessive embolization is not expected. Other materials including coils, auxiliary stents, and balloons can be considered if necessary. Lastly, in preoperative or intraoperative evaluation, a multistage embolization strategy is feasible if complete embolization could not be fulfilled by a single operation. Patients 6, 7, 9, 14, and 23 (described in Table 2) received two stages of treatment to avoid probable complication risks following a single complete occlusion (Details are described in the discussion section).

### Outcomes

**Figure 2:**
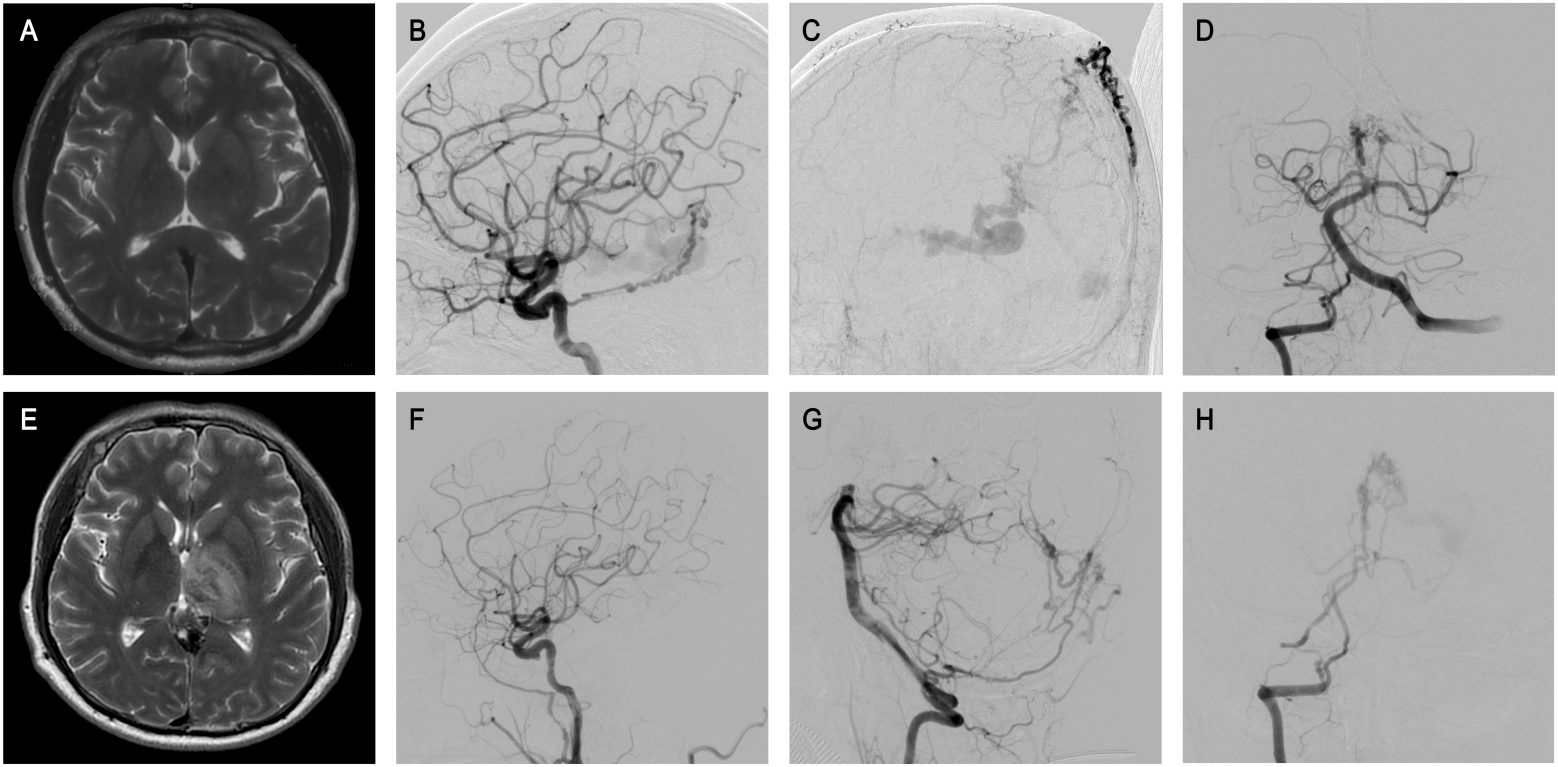
The patient 33 presented that physical examination revealed intracranial vascular malformation for 3 months. **(A)** Preoperative MRI showed no obvious abnormalities. **(B–D)** ICA and VA, and ECA angiography revealed GDAVF feeded by the bilateral MMAs, OAs, MHTs, and right PMA, left IMA, left MPChA. **(E)** Half a month later, MRI sacan showed abnormal signals in bilateral thalamus area in T2, which was the manifestation of venous infarction. **(F–H)** Postoperative angiographic image. Postoperative angiogram images showing complete obliteration of the GDAVF **(F, G)**. Right VA arteriography suggested the intravenous outflow time of contrast agent was delayed **(H)**.

In all 31 patients, only two declined to receive interventional treatment. Beyond that, 29 patients received endovascular embolization, and no perioperative death occurred. Seventeen patients had no observable symptoms after treatment, and eight patients had mild symptoms without any impacts on normal life and work. However, only four patients showed slight improvement after treatment when compared with the preoperative level: two patients' poor outcomes may be attributed to initial severe intracranial hemorrhage stroke (patients 10 and 29), and another five patients suffered severe cognitive disorders, all of which resulted in poor prognosis. Notably, only one patient developed cognitive dysfunction within 2 weeks of surgery, and postoperative magnetic resonance images suggested thalamic infarction (patient 27 described in Figure 2).

Obviously, cognitive impairment could be an important factor affecting prognosis, and occurred in eight of the 31 patients before treatment. Specific symptoms, including memory impairment, attention deficit, and emotional lability, were prominent manifestations. Among the eight patients with varying degrees of cognitive impairment, one was treated conservatively with an unfavorable prognosis, and seven underwent interventional treatment. Cognitive impairment persisted in the seven treated patients, suggesting that the long course of the disease may have resulted in pathological irreversibility. The median duration of long-term follow-up was 24 months (ranging from 12 to 96 months). All patients were followed up with imaging examination involving MRI and DSA, or through telephone for follow-up, the outcomes' grades according to modified Rankin Scale (mRS) were summarized in Table 1.

## Discussion

From the pathophysiological and therapeutic perspective, there are unique angioarchitectural characteristics of aGDAVFs, which are mainly derived from their location and venous drainage. Interesting points are discussed to comprehensively understand this nidus and to share the optimal treatment options.

### Definition and general description

AGDAVFs are the most complex type of DAVF, and are difficult to manage because of their deep location and unusual vascularity. Except for some case reports or series, aGDAVFs have seldom been reported as a separate type of DAVF, and there was no specific management guidance on aGDAVFs from the endovascular treatment perspective (6). In Lawton's work, as a type of tentorial DAVFs, seven cases of DAVF at the Galenic region were reported, which were removed by surgery (7). A few cases that were not clearly defined were also recorded in previous documents (8, 9). As Lawton described, GDAVF were located in the midline at the posterior margin of the tentorial incisura, and the vein of Galen entered the anterior falcotentorial junction. Supratentorial and/or infratentorial venous drainages could be found. Notably, to define the lesion in this area and describe its characteristics more accurately, it would be of significance to make sure the cases are pure DAVFs but not vein of Galen arteriovenous malformations (VGAMs). VGAMs typically occur in young children and are described as occurring from the 6th and 8th to 11th weeks of gestation (10). Depending on the occurrence or absence of venous anatomy development at these stages, venous drainage from VGAMs is variable, with many potential outflow routes. Lasjaunias et al. described this lesion in detail and established various classifications of VGAMs (11–13). However, since these defects are so rare, malformations that drain into the Galen vein or nearby venous system, as well as those with Galen vein thickening and venous lake formation, were often described as VGAMs in the literature, without specifying the venous characteristics of the DAVF in the Galen region. Some clear descriptions about its characteristics are listed as follows: (1) The supply by meningeal arteries, especially from the ECA, is usually dominant, and simultaneous suppliers from multiple systems are common, as mixed pial and dural feeders are often found. (2) The number of fistulas varies greatly, and the majority of patients may have numerous fistulas and are distributed distinctively. (3) Abnormal venous enlargement with venous lake formation are common angiographic findings.

### Anatomy of the Galen area and its tributaries

The Galen vein is located in an important anatomical structure of the quadrigeminal cistern in the deep brain, which lies between the atrial portion of the paired choroidal fissures (14, 15). Five walls (one superior, one inferior, one anterior, and two lateral) contribute to the configuration of the cistern (4).

The roof (superior wall) and the floor (inferior wall) are formed by the lower surface of the splenium of the corpus callosum and the super-oventral portion of the cerebellum respectively. The anterior wall is the corpora quadrigemina, also known as two paired superior and inferior colliculus, and the pineal body lies between the paired superior colliculi. The two symmetrical lateral walls are composed of the fornix crus and the occipital cortex. The Galen vein drained into the straight sinus. Accordingly, we call this area, including the vein of Galen with its draining veins and branches, as well as the surrounding structures, the region of Galen.

Vein tributaries of the Galen region are complex (16, 17). The pineal and tectal veins drain into anterosuperior aspect of the vein of Galen. The SVV and the PCV or SCV, encountered from posterior to anterior, drain into its posteroinferior side. The ICVs are the main anterior veins, and they join to form the vein of Galen. Meanwhile, from the two lateral sides, the basal vein (BV) drains into the Galen vein. Remarkably, the ICVs and BVs are recognized as the major origin of the Galen vein.

Since these veins are closely related to the function of surrounding brain regions, thrombosis formation and embolic sacrifice, which decreased the potential for collateral formation, may lead to the serious consequences (16). In line with this, cortical veins reflux and venous hypertension would occur, as the ICV and BV reverse their flows and drain backwards when the normal drainage pathways are interrupted. Moreover, the straight sinus is the unique normal outlet of Galen vein, and its patency is often related to the function of the deep venous system (DVS) (3). Interventional treatment should ensure that the DVS is unblocked as much as possible, and the sacrifice of any vein should be considered carefully, especially in the straight sinuses (18, 19).

### Characteristic clinical signs

In recent years, with the promotion of national health awareness, the number of asymptomatic patients with DAVF is gradually increasing. Five patients in our study had no warning signs prior to their hospital visit (patients 1, 19, 24, 27, and 30), and anomalies were incidentally discovered through physical examination. However, the majority of patients still have symptoms. Currently, the presentations of intracranial galenic DAVF could be generalized into three categories: (1) Symptoms of pulsatile tinnitus, intracranial vascular murmur and ophthalmological phenomenon causing by the increased dural sinus drainage; (2) ICH and subsequent neurological deficits; and (3) Non-hemorrhagic neurological deficits (NHNDs), which need to be further recognized. Of course, most of these symptoms are strongly related to drainage patterns, especially the presence or absence of cortical veins drainage (20, 21).

Notably, in the present work, we mainly emphasize the description of progressive dementia, which is an under-recognized sign of DAVFs. Thanks to the improvement in imaging and analytical techniques, cortical venous hypertension induced thalamic dementia could be correctly diagnosed and treated, though DAVFs are one of a number of conditions that can cause thalamic dementia (22). However, patients who suffer the deep venous system thrombosis, infectious and toxic insults, metabolite deposition and osmotic myelinolysis, and even occupying tumors in this region may share similar symptoms as the pathological conditions of the thalami (5, 23). Thus, a thorough clinical history may provide strong clues when distinguishing these numerous etiologies, for various pathogenic factors may have certain characteristics. Furthermore, neuroimaging techniques such as MRIs can further enhance the identification of these possible causes. In most situations, progressive dementia associated with aGDAVF is usually visible on an MRI as bilateral thalamic high signal on T2/FLAIR sequences (24–26).

However, variability in these signal characteristics is not uncommon (27, 28). The complex or unclear image signals may lead to an unclear diagnosis, which may cause delays in treatment. More importantly, just as in our study, not all patients with obvious dementia symptoms have typical imaging findings. Thus, we suggest that the use of MRIs and angiographies such as DSA are both necessary for undetermined progressive dementia. Our results suggest that cognitive dysfunction may be very hard to reverse, so timely diagnosis and effective treatment are necessary to stop the deterioration process.

### Endovascular intervention

According to the architecture and hemodynamic characteristics of the DAVF in the Galenic region, we summarized applicable embolization strategies that were optimal to enhance their safety and effectiveness, as well as to reduce complications.

Firstly, DAVFs with pial arterial feeders, which are also called mixed DAVFs, have rarely been documented (29). However, the risk caused by pial artery supplied fistula during the endovascular treatment might not be fully appreciated. Since the pial feeding arteries are often tortuous, tenuous, and fragile, the process of super-selective catheterization is difficult, with increasing bleeding risk (29–31). An effective strategy is to deal with these feeders first before the fistula embolization is suggested. As conventional, multi-stage endovascular embolization is acceptable when one single complete embolization is predicted to increase operational risk. Furthermore, skillful and gentle handling with optimal materials is highly advisable, avoiding hemorrhagic complications from an interventional operation.

Second, the options of approaches and materials are commonly considered when treating DAVFs by endovascular intervention, while transarterial fluid embolization is primarily recommended (32–34). Fluid agents including Onyx, NBCA (n-butyl cyanoacrylate) and Glubran were mainly used in our cases. The characteristics of these liquid embolic agents are briefly summarized as follows: (1) Onyx glue is an ideal material for endovascular embolization in the treatment of dural arteriovenous fistula. Before using it, Onyx needs to be shaken with dimethyl-sulfoxide (DMSO) and tantalum powder for about 20 min to form a polymer suspension. Then Onyx gradually forms embolization from the periphery to the inner core, which may take about 10 min. Onyx has a better mechanical filling effect, so it is suitable for embolization of more complex fistulas. At the same time, Onyx releases less heat in the embolization process, so the probability of postoperative inflammation is lower. (2) The embolization of NBCA formed in the blood vessel is permanent, and it has a beneficial effect on the embolization of large blood vessels with its outstanding mechanical strength. However, before injection, the microcatheter needs to be rinsed with a non-ionic 5% glucose solution to prevent premature intraductal embolization. At the same time, the microcatheter should be removed in time after glue injection to prevent the hardening substance from embolizing in the microcatheter. All of these procedures require a skilled surgeon and the help of a qualified assistant. On the other hand, for delicate blood vessels, NBCA embolization agents are difficult to penetrate completely. Moreover, when complex lesions require multiple embolizations, it cannot be achieved with NBCA alone. (3) Glubran glue (NBCA-MS, 2-methylstyrene-α-isobutyl cyanoacrylate) is a kind of adherent liquid embolization material produced on the basis of the original NBCA. Its polymerization time is extended from 15–40 s (NBCA glue) to 60–90 s, which provides a sufficient time window for full and uniform embolization. It also avoids the risk of microcatheter adhesion caused by premature polymerization, resulting in difficult catheter removal and even bleeding.

Compared with Onyx, Glubran is thought to diffuse faster and regurgitate less during embolization, which allows easier catheter removal. In general, to achieve perfect embolization and minimize the occurrence of complications with liquid embolic agents, the selection of injection-related vessels, the concentration of glue, and the speed of injection are crucial. In addition, to prevent the agents migrating into the perforating vessels of the thalamus, the rapid polymerization property of the agents should be considered (34).

Advances in materials science and technology have encouraged us to eliminate the culprit both in symptoms and anatomy. In addition, the pressure cooker, balloon-assisted embolization, and multi-plug techniques were widely applied to enable the liquid agent to flow forward and penetrate without reflux (35–38). All these measures could be taken to achieve the most distal embolization without accident complication.

Third, complete fistula embolization should be performed while conserving the patency of the DVS as much as possible. Excessive venous occlusion could be the main reason for unexpected complications such as progressive thrombosis and venous-related infarcts post-embolization (6). As the cautionary case in Figure 2 shows, although there were no inappropriate steps throughout the embolization procedure, the postoperative angiography suggested the intravenous outflow time of contrast agent was delayed, which might indicate excessive embolization. The MRI scan showed bilateral thalamus infarction, which may explain the cognitive dysfunction of the patient. On the other hand, occlusion of the venous component that is too early may result in bleeding. When the sacrifice of a vein is inevitable, a clear understanding of vascular anatomy and draining patterns is strongly needed. Any haphazard attempts at venous sacrifice may result in serious complications. The straight sinus should be protected as much as possible, ensuring the venous outflow of Galen is unobstructed. Although it is unclear whether it may be beneficial to try to unblock the straight sinus, interventional occlusion is not encouraged if it is unobstructed before surgery.

Lastly, due to extremely tortuous feeders, super-selective catheterization failing to reach a fistula is possible. Thereby, pathway creation through a transosseous approach may be a valuable strategic option to overcome this limitation (39–42). In very specific cases, as we described in Figure 1, when endovascular access is not possible, direct external occipital protuberance puncture was shown to be feasible and effective. With the development of related assistive techniques including intraoperative navigation, ultrasound-guided procedures, and three-dimensional reconstructed imaging, the safety of such operations would be greater.

## Conclusion

Our work recorded the largest number of aGDAVF cases to date, emphasizing the unique angioarchtecture as an individual entity. The symptoms of progressive dementia need to be further recognized. With a high risk of aggressive clinical course, early diagnosis and timely intervention is crucial for a good prognosis. Pial-first transarterial embolization is suggested. Excessive embolization with deep vein system obstruction is not recommended. Surgical path creation is encouraged when conventional approaches cannot not be undertaken satisfactorily. Based on the detailed understanding of anatomical architectures and drainage patterns, aGDAVFs can be embolized safely and effectively by judicious endovascular interventional strategies.

## Data availability statement

The raw data supporting the conclusions of this article will be made available by the authors, without undue reservation.

## Ethics statement

The studies involving human participants were reviewed and the study was approved by Ethics Committee of Xuanwu Hospital of Capital Medical University. The patients/participants provided their written informed consent to participate in this study.

## Author contributions

TT analyzed the data, drafted the manuscript, and arranged ideas. ZS collected the data and contributed to the analyzation. YM revised the manuscript and designed the key points of writing. CY participated in data collection. CH, GL, TH, LS, and PH guided the operation and controlled the operation steps. PZ, MY, and HZ performed operations, designed the concept, and approved the paper for publication. All authors made critical revisions of the manuscript and reviewed the final version.

## Funding

This study was funded by National Natural Science Foundation of China (Award Number: 82101460).

## Conflict of interest

The authors declare that the research was conducted in the absence of any commercial or financial relationships that could be construed as a potential conflict of interest. The handling editor LM, declared a shared parent affiliation with the authors at the time of review but no collaboration or relationships with the authors.

## Publisher's note

All claims expressed in this article are solely those of the authors and do not necessarily represent those of their affiliated organizations, or those of the publisher, the editors and the reviewers. Any product that may be evaluated in this article, or claim that may be made by its manufacturer, is not guaranteed or endorsed by the publisher.

## Abbreviations

ECA: external carotid artery
ICA: internal carotid artery
VA: vertebral artery
MMA: middle meningeal artery
OA: occipital artery
STA: superficial temporal artery
APA: ascending pharyngeal artery
PAA: posterior auricular artery
IMA: internal maxillary artery
MHT: the meningohypophyseal trunk
OphA: ophthalmic artery
ACA: anterior cerebral artery
MCA: middle cerebral artery
AChA: anterior choroidal artery
PMA: posterior meningeal artery
PCA: posterior cerebral artery
SCA: superior cerebellar artery
MPChA: medial posterior choroidal artery
LPChA: lateral posterior choroidal artery
ICV: internal cerebral vein
BVR: the basal vein of Rosenthal
IJV: internal jugular veinc
DVS: deep venous system
SVV: superior vermian vein
PCV: precentral cerebellar vein
SCV: superior cerebellar vein
aGDAVF: Adult Galenic Dural Arteriovenous Fistula
ICU: intensive care unit
mRS: modified Rankin Scale
NHNDs: Non-hemorrhagic neurological deficits.
